# JAK2 V617F polycythemia vera and essential thrombocythemia: dynamic clinical features associated with long-term outcomes

**DOI:** 10.1038/s41408-022-00646-0

**Published:** 2022-04-08

**Authors:** Léa Sureau, Caroline Buors, Jean-Christophe Ianotto, Françoise Boyer, Aline Tanguy-Schmidt, Lydia Roy, Emilie Cayssials, Laura Cailly, Jean-Claude Chomel, Aurélie Chauveau, Corentin Orvain, Olivier Mansier, Dana Ranta, Margot Robles, Emmanuel Gyan, Olivier Hérault, Stanislas Nimubona, Tony Marchand, Eric Lippert, Jérémie Riou, Valérie Ugo, Damien Luque Paz

**Affiliations:** 1 CHU Angers, Laboratoire d’Hématologie, Angers, France; 2 Univ Angers, Nantes Université, CHU Angers, Inserm, CNRS, CRCI2NA, F-49000 Angers, France; 3 Fédération Hospitalo-Universitaire “Grand Ouest Against Leukemia” (FHU GOAL), Angers, France; 4 CHU Brest, Laboratoire d’Hématologie, Brest, France; 5 CHU Brest, Service d’Hématologie Clinique, Brest, France; 6 CHU Angers, Service des Maladies du Sang, Angers, France; 7 Hôpital Universitaire Henri Mondor, Service Hématologie Clinique, APHP & UPEC, UFR de Santé, Créteil, France; 8 CHU Poitiers, Service d’Oncologie Hématologique et Thérapie Cellulaire, Poitiers, France; 9 CHU Poitiers, Service de Cancérologie Biologique, Poitiers, France; 10 CH de Cornouaille, Laboratoire d’Hématologie, Quimper, France; 11 CHU de Bordeaux, Laboratoire d’Hématologie et Université de Bordeaux, Inserm U1034, Bordeaux, France; 12 CHU Nancy, Hématologie Clinique, Nancy, France; 13 CH Périgueux, Hématologie Clinique, Périgueux, France; 14 CHU Tours, Service d’Hématologie et Thérapie Cellulaire, CNRS EMR 7001 LNOx, université de Tours EA7501, Tours, France; 15 CHU Tours, Service d’Hématologie Biologique, CNRS EMR 7001 LNOx, université de Tours EA7501, Tours, France; 16 CHU Rennes, Service d’Hématologie Clinique, Rennes, France; 17 INSERM U1236, Faculté de Médecine, Université Rennes 1, Rennes, France; 18 CHU Angers, Département de Biostatistiques et de Méthodologie, DRCI, Angers, France; 19 Univ Angers, CHU Angers, Inserm, CNRS, MINT, SFR ICAT, Angers, France

**Keywords:** Myeloproliferative disease, Prognosis

**Dear Editor**,

Myeloproliferative neoplasms (MPN) are acquired clonal hematopoietic stem cell disorders characterized by abnormal proliferation leading to accumulation of mature blood cells. They comprise polycythemia vera (PV), essential thrombocythemia (ET), and primary myelofibrosis (PMF). A somatic *JAK2*V617F mutation is found in almost all cases of PV and ~60% of ET and PMF resulting in the constitutive activation of JAK-STAT pathway. PV and ET are the most indolent MPN with a median survival over 10 and 15 years, respectively [1]. Nevertheless, overall survival is reduced by two major complications: an increased risk of both thrombosis and hemorrhage and, in the long-term, a risk of evolution to myelofibrosis (MF) or accelerated phase (myelodysplastic syndrome (MDS) or acute myeloid leukemia (AML)). Prognostic classifications in ET and PV are based on characteristics at diagnosis: age, leukocyte count, history of thrombosis, and the presence of *JAK2*V617F [2–4]. However, no dynamic prognostic systems have been proposed for these chronic diseases followed for decades.

In the present work, we aimed to evaluate the prognostic relevance of defining an early clinical worsening at 3 years of follow-up in a cohort of *JAK2*V617F-mutated ET or PV patients. For this purpose, we analyzed data from the prospective JAKSUIVI study that was conducted from 2007 to 2013 in six French University Hospitals. In this study, 191 *JAK2*V617F-positive PV or ET patients were enrolled at the time of diagnosis and the worsening status was defined after 3 years of follow-up by at least one of the following criteria: (i) leukocytosis >12 G/L or presence of immature granulocytes >2% or erythroblasts >1%; (ii) anemia (hemoglobin <12 g/dL in a woman or <13 g/dL in man) not related to treatment toxicity; (iii) thrombocytopenia (platelet count <150 G/L) not related to treatment toxicity; (iv) onset of splenomegaly or progression of pre-existing splenomegaly; (v) thrombocytosis despite cytoreductive therapy. The working hypothesis of JAKSUIVI study was to test at 3 years of follow-up, a possible association between *JAK2*V617F allele burden evolution and clinical worsening (Supplemental Table S1). Herein, we report the long-term prognostic impact of this worsening criterion with a median cohort follow-up of 10 years (Fig. 1A).

**Fig. 1:**
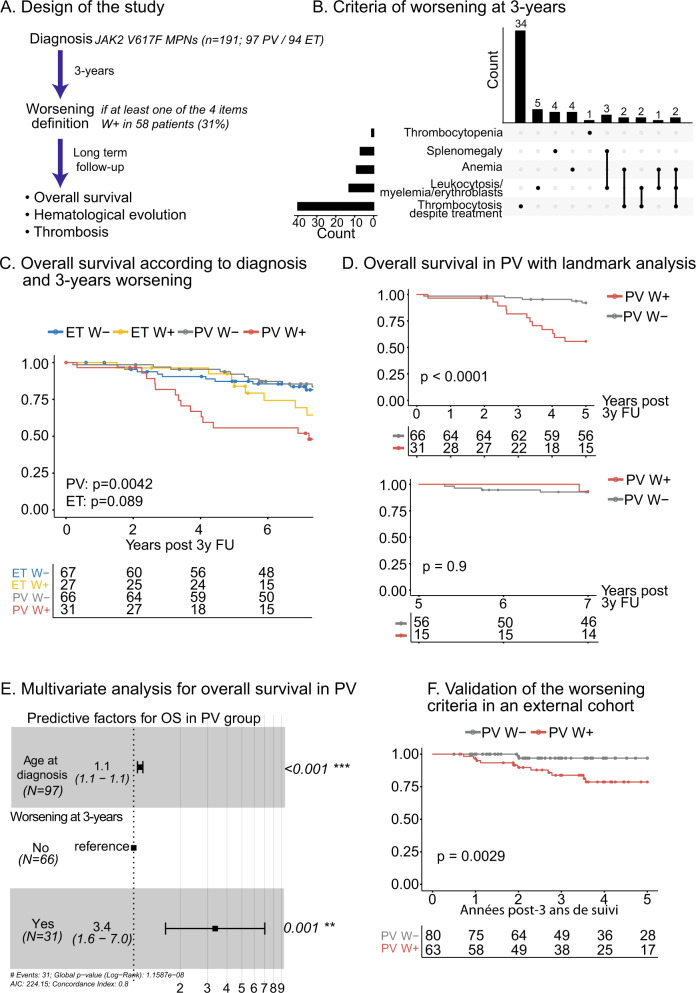
Worsening at 3 years and long-term prognosis. **A** Flow-chart representing the design of the JAKSUIVI study. **B** Distribution of items for the worsening criteria at 3 years of follow-up. **C** Kaplan–Meier curve represents overall survival according to the diagnosis (ET: essential thrombocythemia, PV: polycythemia vera) and the worsening status (W+: worsened at 3 years, W−: not worsened). Starting time t0 was the 3 years (3 y) follow-up (FU). **D** Kaplan–Meier curves for the landmark analysis of overall survival in PV patients. The top plot represented the follow-up of the five first years after the 3-year evaluation and the bottom plot represented the follow-up from 5 to 7 years after the 3-year evaluation with a t0 at 5 years. **E** Forest plot representing the results of multivariate Cox analysis for overall survival in PV. **F** Kaplan–Meier curve represents overall survival according to the worsening status in the validation cohort.

After 3 years of follow-up, 58/191 (30.4%) patients met worsening criteria (W+) with a similar proportion between PV and ET (Table 1). For 48/58 (82.8%) of these patients, only one criterion for W+ definition was present, the most common being persistent thrombocytosis despite hydroxyurea therapy present in 40 patients (18 PV and 22 ET patients) (Fig. 1B). The main reasons for the 51 deaths observed in the cohort were: other neoplasms (13.7%), thrombosis (9.8%), cardiac failure (9.8%), secondary AML (9.8%), infection (5.9%). These data were missing for 37.3% of patients. Kaplan–Meier analysis showed that W+ status was predictive of decreased overall survival with significance in PV patients (*p* = 0.0042, Fig. 1C). The univariable analysis also revealed that age at diagnosis (*P* < 0.001) and leukocyte count (*P* = 0.042) but not gender, history of thrombosis, or *JAK2*V617F allele burden were associated with reduced overall survival in PV (Supplemental Table S2). As suspected, the proportional hazard assumption of death events was not valid among the groups W+ and W−. Thus, we performed landmark analyses that highlighted different profiles (Fig. 1D). Indeed, the worsening status was able to distinguish events occurring during the 5 years following the worsening evaluation but not after (Fig. 1D). Finally, applying a multivariable Cox model with a manual backward variable selection, which minimizes the Akaike criteria, we confirmed that both worsening status (HR: 3.4; *P* = 0.001) and age at diagnosis (HR: 4.9; *P* = 0.003 for age >60 years, Fig. 1E) were associated with reduced overall survival in PV patients. Furthermore, the W+ status significantly predicted overall survival independently of the prognostic classification proposed by Tefferi et al. [2]. (HR: 2.9; *P* = 0.004 for worsening status and HR:4.7; *P* = 0.002 for high risk vs low risk; Supplemental Fig. S1).

**Table 1:** Characteristics of the whole cohort.

	Global cohort	PV	ET
Participants (*n*)	191	97	94
Age at diagnosis (y)	65 [23; 90]	64 [24; 90]	65 [23; 85]
Gender (M/F)	96 (50.26%)/95 (49.74%) R: 1.01	56 (57.73%)/41 (42.27%) R: 1.37	40 (42.55%)/54 (57.45%) R: 0.74
*At diagnosis*			
Biological data at diagnosis (median [IQR])			
Hemoglobin (g/dL)	15.6 [11.2;25]	17.8 [12.2;25]	14.6 [11.2;17.7]
Hematocrit (%)	46.9 [33.5;72.8]	53.6 [40.8;72.8]	43.7 [33.5;52.7]
Platelets (G/L)	618 [135;1378]	514 [135;1262]	698 [392;1378]
Leukocytes (G/L)	9.8 [4.4;21.3]	10.7 [5.5;21.3]	8.95 [4.4;18]
Neutrophils (G/L)	6.9 [1.15;18.5]	8 [1.15;18.5]	6.2 [2.8;15.35]
JAK2*V617F* allele burden (%)	24.24 [1.26; 94.96]	39.81 [1.26; 94.96]	15.3 [1.66; 68.36]
At 3 y FU			
*Biological data at 3 y FU (median [IQR])*			
Hemoglobin (g/dL)	13.9 [8.9; 17.9]	14.1 [8.9; 17.9]	13.7 [10.8; 17.3]
Hematocrit (%)	41.7 [29.8; 55.1]	42.8 [29.8; 55.1]	40.85 [32.2; 52.8]
Platelets (G/L)	350 [98; 1110]	287 [98; 712]	386.5 [149; 1110]
Leucocytes (G/L)	6.2 [2.2; 38.2]	6.2 [2.6; 38.2]	6.5 [2.2; 18.5]
Neutrophils (G/L)	4.3 [1.08; 16]	4.2 [1.08; 16]	4.45 [1.3; 15.8]
JAK2*V617F* allele burden (%)	15.02 [0; 91.37]	24.37 [0.12; 91.37]	10.075 [0; 80.25]
Absolute change in allele burden	−5.1 [−81; +36.98]	−9.82 [−81; +36.98]	−2.125 [−31.7; +25.1]
Relative change in allele burden	−21.088 [−100; +578.659]	−33.56 [−99.255; +578.659]	−16.2 [−100; +233.65]
Decreased allele burden (absolute ≥−10% or relative ≥−50%)	77 (40.3%)	50 (51.5%)	27 (28.7%)
*Recorded events at inclusion (n, %)*			
Splenomegaly (clinical or volume >12 with ultrasound)	52 (27.2%)	36 (37.1%)	16 (17%)
Thrombosis history	64 (33.5%)	30 (30.9%)	34 (36.2%)
Hemorrhage history	16 (8.4%)	8 (8.3%)	8 (8.5%)
Worsening status at 3 y FU (*n*, %)	58 (30.4%)	31 (32%)	27 (28.7%)
1 event	48 (82.8%)	27 (87.1%)	21 (77.8%)
2 events	8 (13.8%)	4 (12.9%)	4 (14.8%)
3 events	2 (3.4%)	0 (0%)	2 (7.4%)
*Long-term follow-up*			
Follow-up time in years (median [IQR]))	10.6 [3; 14.6]	10.5 [3; 14.1]	10.6 [3.3; 14.6]
Thrombosis	35 (18.3%)	19 (19.6 %)	16 (17. 0%)
Transformation	24 (12.6%)	12 (12.4%)	12 (12.8%)
Including secondary MF	14 (7.3%)	6 (6.2%)	8 (8.5%)
Including secondary LAM	10 (5.2%)	6 (6.2%)	4 (4.3%)
Including secondary MDS	2 (1%)	1 (1%)	1 (1.1%)
Deaths	51 (26.7%)	31 (32%)	20 (21.3%)

*FU* follow-up.

Although the worsening status did not significantly affect overall survival in ET patients, we found a higher risk of death after 5 years post evaluation applying the same landmark analysis than in the PV group (*P* = 0.071, Supplemental Fig. S2). In multivariable analysis, age at diagnosis and a history of thrombosis were significant predictive factors of reduced survival (Supplemental Fig. S3).

We then studied the impact of worsening at 3 years on hematological transformation into either MDS, MF, or AML with the same methodology. In ET patients, a W+ status was predictive of an increased risk of hematological transformation 5 years or more after worsening (*P* = 0.00028, Supplemental Figs. S4, and S5 for PV). Finally, we found no significant association between the worsening criteria and thrombotic events in either ET (*P* = 0.52) or PV patients (*P* = 0.82).

To date, prognostic evaluation of PV and ET is assessed at diagnosis based on age, blood counts, and history of thrombosis [5]. In the present work, we propose an early and easy-to-identify worsening criterion evaluated at 3 years of follow-up, associated with reduced survival in PV, independently of other classical prognosis markers. To validate this finding, we applied the worsening criteria at 3 years to an independent multicentric cohort of 147 PV patients (from FIMBANK network, details in Supplemental data) and found similar results as patients with a W+ status at 3 years displayed a higher risk of death during the first 5 years (Fig. 1F, *p* = 0.0029), but not afterward (*P* = 0.41, data not shown). Therefore, in PV patients, both initial and validation cohorts, the worsening status was associated with a higher risk of death during the first 5 years post-assessment (i.e., 3–8 years after diagnosis), but not in a longer-term. It is possible that repeating the worsening evaluation during follow-up may improve the prognostic assessment of late events in PV. In contrast, for ET patients, we observed that the W+ status at 3 years was associated with events occurring only after 8 years of follow-up, with a significant difference for hematological transformation and a trend for overall survival. These results may be explained by the different prognostic course of ET compared with PV, with a late excess of mortality during follow-up and a lower rate of short-term fibrotic or leukemic evolution [1, 6–8].

Additional mutations can be found in MPN, some of which are associated with a significantly altered outcome [9]. This has been mostly studied in PMF, which presents with a more complex molecular landscape with some mutations recently incorporated into a prognostic scoring system [10]. In PV and ET, a prognostic score has also been recently published based on the identification of adverse mutations in *SRSF2* for PV and in *SRSF2*, *SF3B1*, *U2AF1*, and *TP53* in ET [11]. However, *SRSF2* mutations occurred in only 1–3% of PV patients [9, 12], thus limiting the number of patients benefitting from this prognostic assessment. At the last ASH meeting, the potential prognostic impact of repeated molecular evaluations in PV and ET using next-generation sequencing (NGS) has been reported [13]. Emerging mutations, which were detected in 29% of patients with a median interval between NGS evaluation of 1.6 years, were associated with reduced overall survival. This suggests that a dynamic scoring system can also include additional mutations. This should be investigated in future prospective studies to determine in which patients and how often such evaluation should be performed.

To conclude, we propose a simple and easy-to-use worsening criterion allowing identification of patients at high risk at 3 years of follow-up. These results pave the way for a new dynamic prognostic system in PV that will need validation for any follow-up time. Indeed, the re-evaluation of prognosis during the follow-up is crucial in such chronic diseases, as it could influence patient management and therapy.

## Reporting summary

Further information on research design is available in the Nature Research Reporting Summary linked to this article.

## Supplementary information

Supplemental data

Reporting summary checklist
