# Supplementary material for: JAK2 V617F polycythemia vera and essential thrombocythemia: dynamic clinical features associated with long-term outcomes
Source: Blood Cancer J. 2022 Apr 8;12(4):56. doi: 10.1038/s41408-022-00646-0 (PMC8993923; doi:10.1038/s41408-022-00646-0)
Supplement: Supplementary file 1 — Supplemental data [file 41408_2022_646_MOESM1_ESM.doc]

**Supplemental data**

**JAKSUIVI study**

JAKSUIVI cohort has included newly diagnosed PV and ET patients, with the aim to correlate the *JAK2*V617F allele burden (%V617F) at diagnosis before any cytotoxic therapy, the %V617F 3 years later, and the variation of the *JAK2*V617F allele burden (ΔV617F) between y0 and y3 with clinical evolution. Primary or secondary myelofibrosis and secondary leukemia were not included. Five centers (Angers, Brest, Poitiers, Rennes and Tours University Hospitals) participated in the study. They respectively included 70, 69, 26, 19 and 7 patients.

The inclusion criteria were: i) patient with polycythemia vera or essential thrombocythemia according to the WHO classification (Vardiman et al, 2008), ii) *JAK2*V617F positive, iii) diagnosed three years (+/- 4 months) before, iv) DNA collection at diagnosis and before any cytotoxic therapy, v) signature of informed consent approved by the local institutional review board (IRB Ouest 6). Also, exclusion criteria were identified: i) diagnosis of PMF, or acute leukemia at diagnosis, ii) age before 18 years-old, iii) patient unable to sign informed consent. Biological data including *JAK2*V617F allele burden were evaluated at diagnosis and 3 years later. At this 3 years follow-up point, clinical evolution was assessed in order to define the worsening status (W+ or W-).

**Validation cohort**

We set up a cohort to validate the results of this study, including 147 patients diagnosed with PV. This validation cohort was extracted from the FIMBANK database supported by the FIM and funded by the French Institut National du Cancer (INCa). All patients are over 18 years old have given informed consent for this data collection and use in translational studies.

These 147 patients were included by 12 of the 28 French hospitals participating in the FIMBANK project. They were from Angers (44), Bordeaux (22), Nancy (20), Brest (20), Périgueux (11), Dijon (8), APHP Saint Louis (7), Cholet (5), Le Mans (4), Rochefort (3), Lyon (2) and Lens (1) hospitals. The inclusion criteria in this validation cohort were: i) patient with polycythemia vera according to the WHO classification, ii) with biological data at diagnosis and 3 years (+/- 6 months) after, iii) with clinical assessment at this 3 years FU point, and iv) at least 6 years of follow-up since diagnosis. The median follow-up time in this validation cohort is 6.6 years. 63 patients (43%) were W+ at the 3y FU point and 11 (7%) known as dead at the time of this study.

**Supplemental Table S1:** Clinical and biological characteristics associated with worsening at 3y follow-up estimated by logistic regression

|  | *Relative Risk (IC 95%, p value)* | | |
| --- | --- | --- | --- |
| *Explanatory* | *Global cohort* | *PV* | *TE* |
| Diagnosis (PV vs ET) | 1.11 (0.70-1.62. p=0.627) |  |  |
| Leucocytes > 11 G/L at diagnosis | **2.68 (1.86 – 3.51. p < 0.0001)** | **2.38 (1.37 – 3.48. p = 0.005)** | **3.14 (1.84 – 4.30. p = 0.0004)** |
| Age at diagnosis > 65 yo | 1.48 (0.96 – 2.10. p = 0.075) | 1.08 (0.57 – 1.76. p = 0.77) | **2.10 (1.11 – 3.32. p = 0.026)** |
| Interferon *vs* other myelosupressive at diagnosis | 1.99 (0.74-3.04. p=0.22) | 1.99 (0.69-3.12. p=0.24) |  |
| Myelosuppressive vs no treatment at diagnosis | 1.09 (0.51-2.46. p=0.832) | 0.41 (0.13-1.31. p=0.125) | 2.45 (0.81-9.15. p=0.138) |
| *JAK2*V617F VAF increased or stable for the 3y following diagnosis | **2.12 (1.34-3.10. p=0.003)** | **2.23 (1.25-3.36. p=0.011)** | 2.32 (1.01-4.46. p=0.067) |

**Supplemental Table S2:** Univariate analysis for overall survival and hematological evolution

|  | Univariate p,value Global cohort (n=191) | Univariate p,value PV (n=97) | Univariate p,value ET (n=94) |
| --- | --- | --- | --- |
| **Overall survival** | | | |
| Age at diagnosis | **7.5e-10** | **4.1e-06** | **0.00022** |
| Gender | 0.94 | 0.26 | 0.45 |
| VAF at diagnosis | 0.19 | 0.79 | 0.4 |
| Worsening + | **0.002** | **0.0059** | **0.096** |
| Leucocytes | **0.0028** | **0.042** | **0.067** |
| Thrombosis history | **0.019** | 0.64 | **0.0043** |
| **Hematological transformation** | | | |
| Age at diagnosis | 0.17 | 0.36 | 0.16 |
| Gender | 0.39 | 0.85 | 0.26 |
| VAF at diagnosis | **0.051** | 0.31 | **0.00042** |
| Worsening + | **0.0093** | 0.37 | **0.0061** |
| Leucocytes | **0.0035** | 0.35 | **0.00084** |
| Thrombosis history | 0.68 | 0.48 | 0.99 |


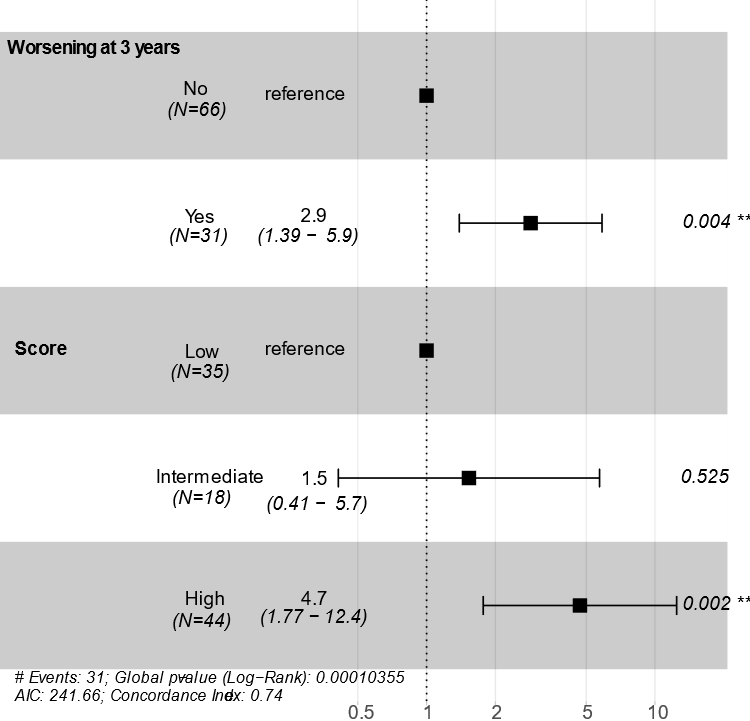


**Supplemental Figure S1:** Forest plot representing results of the multivariate Cox analysis for overall survival in PV with the prognostic score from *Tefferi et al. (Leukemia, 2013)* established at the time of diagnosis.


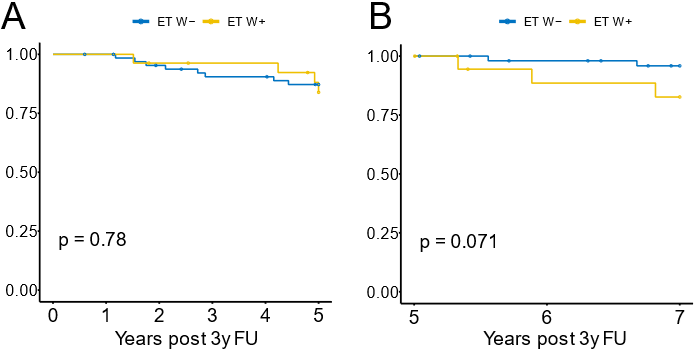


**Supplemental Figure S2:** Landmark analysis for overall survival in ET patients according to worsening (W+). Follow-up (FU) was split into the 5 first years after the worsening evaluation (A) and the late follow-up afterward (B).


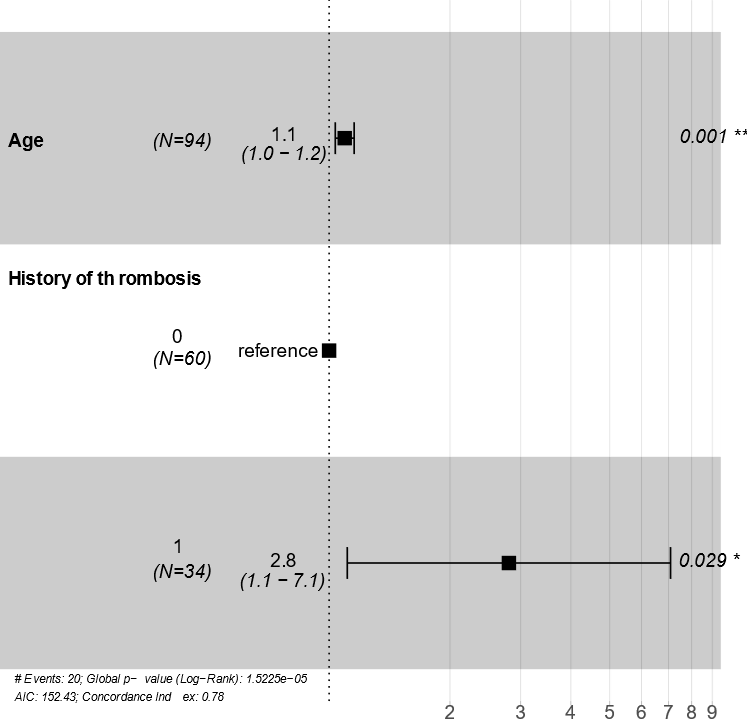


**Supplemental Figure S3:** Forest plot representing results of the multivariate Cox analysis for overall survival in ET patients.


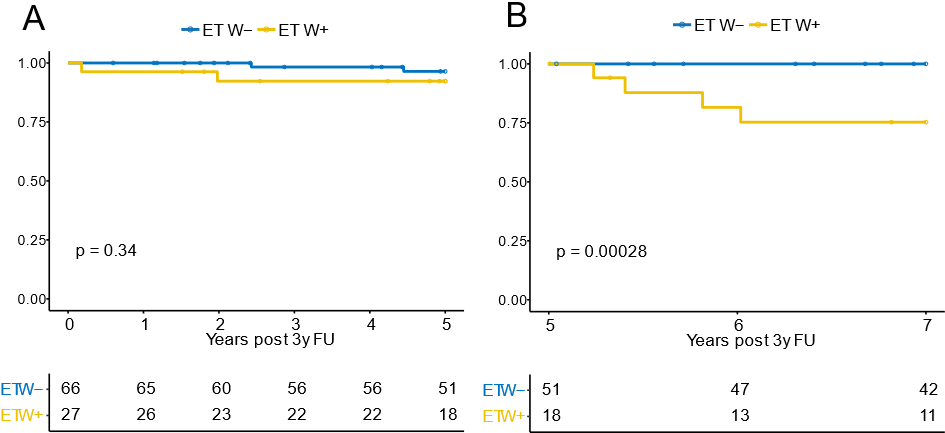


**Supplemental Figure S4:** Landmark analysis for hematological transformation in ET patients according to worsening (W+). Follow-up (FU) was split into the 5 first years after the worsening evaluation (A) and the late follow-up afterward (B).


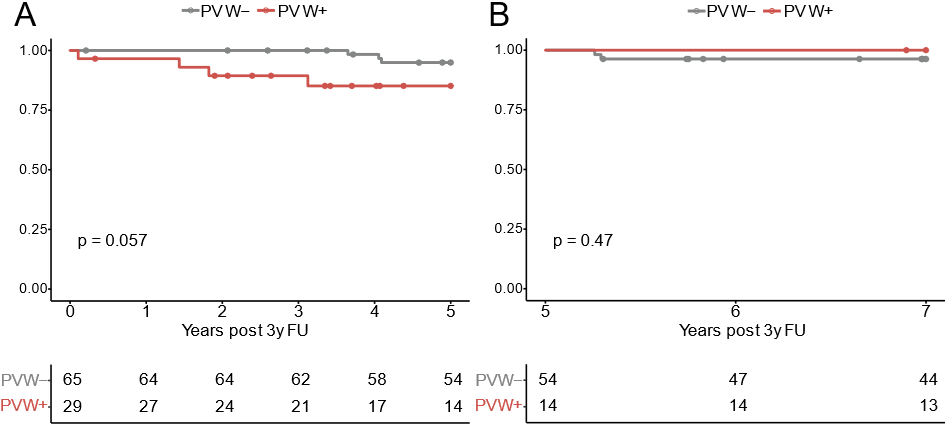


**Supplemental Figure S5:** Landmark analysis for hematological transformation in PV patients according to worsening (W+). Follow-up (FU) was split into the 5 first years after the worsening evaluation (A) and the late follow-up afterward (B).
